# Outcomes with sequential FLT3-inhibitor-based therapies in patients with AML

**DOI:** 10.1186/s13045-020-00964-5

**Published:** 2020-10-08

**Authors:** Musa Yilmaz, Mansour Alfayez, Courtney D. DiNardo, Gautam Borthakur, Tapan M. Kadia, Marina Y. Konopleva, Sanam Loghavi, Rashmi Kanagal-Shamanna, Keyur P. Patel, Elias J. Jabbour, Guillermo Garcia-Manero, Naveen Pemmaraju, Sherry A. Pierce, Issa Ghayas, Nicholas J. Short, Guillermo Montalban-Bravo, Koichi Takahashi, Rita Assi, Ahmad S. Alotaibi, Maro Ohanian, Michael Andreeff, Jorge E. Cortes, Hagop M. Kantarjian, Farhad Ravandi, Naval G. Daver

**Affiliations:** 1 Department of Leukemia, The University of Texas MD Anderson Cancer Center, 1515 Holcombe Blvd, Unit FC4.2012, Houston, TX 77030 USA; 2 Department of Hematopathology, The University of Texas MD Anderson Cancer Center, Houston, TX USA

**Keywords:** FLT3 mutations, Sequential FLT3 inhibitors, Midostaurin, Sorafenib, Quizartinib, Gilteritinib, FLT3-PCR, Low-intensity therapy

## Abstract

**Background:**

Second-generation FLT3-inhibitors (FLT3i) demonstrated single-agent composite CR rates (CRc) of 45–55% in patients with relapsed/refractory (R/R) FLT3-mutated AML in phase II/III trials. However, > 85% of patients treated were prior FLT3i naïve. The response rates to sequential FLT3i exposure remain poorly defined.

**Methods:**

We retrospectively reviewed patients with *FLT3*-mutated AML between November 2006 and December 2019.

**Results:**

In frontline patients treated with a FLT3i (cohort 1), the CRc rates and median overall survival (OS) with the first (*n* = 56), second (*n* = 32), and third FLT3i-based (*n* = 8) therapy were 77%, 31%, and 25%, and 16.7 months, 6.0 months, and 1.4 months, respectively. In patients receiving a FLT3i-based therapy for the first time in a R/R AML setting (cohort 2), the CRc rates and median OS were 45%, 21%, and 10%, and 7.9 months, 4.0 months, and 4.1 months with the first (*n* = 183), second (*n* = 89), and third/fourth (*n* = 29) FLT3i-based therapy, respectively.

In cohort 1, CRc rates with single-agent FLT3i (*n* = 21) versus FLT3i-based combinations (*n* = 19) in second/third sequential FLT3i exposures were 19% versus 42%, respectively. In cohort 2, the CRc rates with single-agent FLT3i (*n* = 82) versus FLT3i-based combinations (*n* = 101) in first FLT3i exposure were 34% versus 53%, respectively, and those with single-agent FLT3i (*n* = 63) versus FLT3i-based combinations (*n* = 55) in second/third/fourth sequential FLT3i exposures were 13% versus 25%, respectively.

**Conclusion:**

CRc rates drop progressively with sequential exposure to FLT3i’s in *FLT3*-mutated AML. In all settings, CRc rates were higher with FLT3i-based combinations compared with single-agent FLT3i therapy in similar FLT3i exposure settings.

## Background

Multiple tyrosine kinase inhibitors (TKIs) have demonstrated clinical activity in *FLT3*-mutated acute myeloid leukemia (AML), including midostaurin, sorafenib, gilteritinib, quizartinib, and crenolanib [1, 2]. First-generation FLT3-inhibitors (FLT3i’s) lack specificity for FLT3 (e.g. midostaurin and sorafenib), while second-generation FLT3 inhibitors (e.g. gilteritinib, quizartinib, and crenolanib) appear to be more potent and specific for FLT3, with fewer off-target effects.

The approval of first-generation FLT3i midostaurin, based on improved overall survival (OS) in the phase III RATIFY trial, established the addition of midostaurin to induction therapy as a standard approach in newly diagnosed *FLT3*-mutated AML [3]. Second-generation FLT3i’s gilteritinib (approved in the USA and Europe for relapsed/refractory (R/R) *FLT3*-mutated AML based on improved OS compared with investigator choice salvage therapy in the phase III ADMIRAL trial) and quizartinib (approved in Japan based on improved OS in R/R *FLT3*-ITD-mutated AML compared with investigator choice salvage therapy in the phase III QuANTUM-R trial) demonstrated single-agent composite complete remission (CRc) rates (CRc = CR + CR with incomplete platelet recovery (CRp) + CR with incomplete count recovery (CRi)) of 45–55% in patients with R/R FLT3-mutated AML [4–7].

A majority (> 85%) of the R/R *FLT3*-mutated AML patients treated in the gilteritinib and quizartinib phase II/III trials were prior FLT3 inhibitor naïve [4–13], a population that is quickly becoming obsolete with increased testing and appropriate widespread addition of midostaurin (or sorafenib where midostaurin is not yet available) to frontline induction in patients with *FLT3*-mutated AML. Subset analysis from the phase II/III trials, in small numbers of R/R *FLT3*-mutated AML patients who had prior exposure to sorafenib or midostaurin and were subsequently treated at R/R presentation with either quizartinib or gilteritinib, demonstrated lower, but still meaningful CRc rates of 25–30% [5, 10]. The benchmark response rates to second and even potentially third FLT3i sequential exposure, to a second-generation FLT3i in patients previously exposed to another prior second-generation FLT3i, and to single-agent FLT3i versus FLT3i-based combinations in R/R FLT3 AML, are important clinical practice and future trial development questions that remain poorly defined. We report our experience with sequential FLT3i-based therapies in patients with *FLT3*-mutated AML treated at our facility.

## Methods

### Patient eligibility

Adults (>/= 18 years) with frontline or R/R FLT3-ITD and/or -D835-mutated AML, who had received at least one FLT3i-based therapy at the University of Texas MD Anderson Cancer Center (UT/MDACC) between November 2006 and December 2019 were eligible. The data cutoff was May 1, 2020. Single-agent FLT3i, FLT3i-based combinations with intensive cytotoxic chemotherapy (CCT) and with low-intensity therapy (LIT) (hypomethylating agent or low-dose cytarabine-based combinations) were included.

The most common FLT3i’s (comprising 95% of total FLT3i exposures) included were sorafenib, midostaurin, quizartinib, gilteritinib, and crenolanib (Additional file 1: Tables S3, S4). A majority of the treatments (87%) were given on clinical trials. All clinical trials utilized are outlined in Additional file 1: Table S1. The study was conducted in accordance with the Declaration of Helsinki. All patients had signed an informed consent form approved by the Institutional Review Board (IRB). Data were collected under MDACC protocols DR09-0223 and PA12-0395 for patients with *FLT3*-mutated AML.

### Study design and objectives

Treatment responses and R/R disease were defined as per modified International Working Group criteria [14]. Our aim is to evaluate the CRc rate and survival (OS, event-free survival (EFS)) with sequential FLT3i-based therapy exposures, and to compare the CRc rates and OS, EFS with single-agent FLT3i’s versus FLT3i-based combinations in similar FLT3i exposure settings. CRc (CR + CRp + CRi) was as previously described in FLT3i phase III studies [6, 7, 15, 16].

Some patients received more than 1 FLT3i-based treatment during the course of their therapy. Each FLT3i-based treatment received was independently analyzed as a FLT3i-based treatment event.

Cohort 1 included patients who received their first FLT3i-based therapy in the frontline setting followed by subsequent FLT3i-based salvages. Cohort 2 included patients who received their first FLT3i-based therapy in salvage, either as the first exposure to a FLT3i in salvage or as a sequential exposure to a FLT3i in salvage. Methodologies for molecular (including multiplex polymerase chain reaction (PCR) analysis for ITD and kinase domain (D835) mutations) and multiparametric flow cytometry (MFC) assessments are in Additional file 1: Table S2**.**

### Statistical methods

Patient characteristics were summarized using median (range) for continuous variables and frequency (percentage) for categorical variables. Categorical variables were compared for significance using the χ2 or Fisher’s exact test, and continuous variables were analyzed using the Wilcoxon rank-sum test. OS was calculated from the date of leukemia therapy to the date of death due to any cause, censored at the last follow-up. EFS was calculated from the date of therapy to the date of disease progression, death due to any cause, or last documented follow-up. Kaplan–Meier method was used to estimate the probability of OS, and log-rank test was used to compare OS and EFS between groups of patients. Statistical analyses were performed in GraphPad and SPSS^©^ (version 24).

## Results

**Fig. 1:**
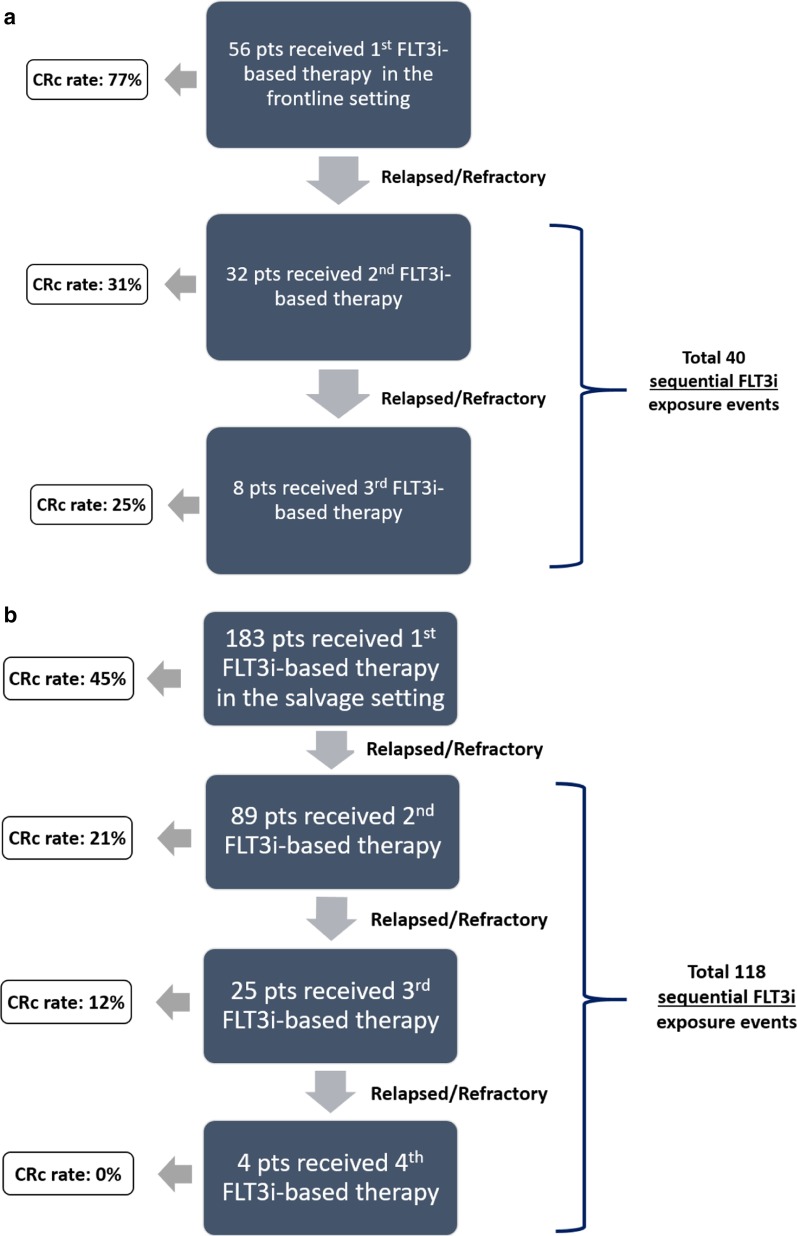
Cohort 1 (**a**) and Cohort 2 (**b**) patient distribution. **a** In cohort 1, 56 patients with newly diagnosed FLT3-mutated AML received induction therapy, and 32 and 8 patients with relapsed/refractory disease received a second or third FLT3i-based therapy, respectively. Overall, 40 subsequent FLT3i exposure events were identified in cohort 1. In cohort 2, 183 patients with relapsed/refractory FLT3-mutated AML received a FLT3-inhibitor based salvage therapy, and 89, 25, and 4 patients with relapsed/refractory disease received a second, third, or fourth FLT3i-based therapy, respectively. A total of 118 subsequent FLT3i exposure events were identified in cohort 2

A total of 239 patients with *FLT3*-ITD- and/or *FLT3*-D835-mutated AML who received FLT3i-based treatments (including single-agent or combination FLT3i therapies) were identified (Table 1). Fifty-six patients received a FLT3i for the first time as a part of their frontline therapy (cohort 1), while 183 patients received a FLT3i for the first time as a part of their salvage therapy (cohort 2) (Fig. 1).

**Table 1:** Clinical characteristics of patients received first FLT3i in the frontline (cohort 1) and relapse/refractory (cohort 2) settings

Baseline clinical features	Cohort 1 (*n* = 56)	Cohort 2 (*n* = 183)
	Median [range], number or positive/tested (%)	
Age, median (range)	62 [22–90]	65 [21–89]
Age ≥ 60	30 (54%)	107 (60%)
Sex, Male	29 (51%)	100 (56%)
sAML	3 (6%)	44 (24%)
Karyotype		
Diploid	36 (64%)	68 (37%)
Monosomy 5/7	3 (5%)	16 (9%)
Trisomy 8	1 (2%)	14 (8%)
11q23-rearrangement	0 (0)	6 (3%)
Miscellaneous	15 (27%)	65 (35%)
Insufficient metaphases	1 (2%)	14 (8%)
Mutations		
NPM1	17/43 (39%)	35/81 (43%)
DNMT3A	10/22 (45%)	21/69 (30%)
RUNX1	3/14 (21%)	9/51 (18%)
TET2	4/14 (29%)	8/31 (26%)
WT1	0/14 (0)	10/49 (20%)
CEBPA	3/20 (15%)	10/69 (14%)
RAS	4/30 (13%)	9/77 (12%)
TP53	0/19 (0%)	4/64 (6%)
ASXL1	1/14 (7%)	6/43 (14%)
IDH1	1/22 (5%)	5/64 (8%)
IDH2	4/22 (18%)	7/70 (10%)
PTPN11	1/18 (6%)	4/64 (6%)
GATA2	0/14(0%)	1/49 (2%)
KIT	1/27(4%)	3/67 (4%)
Frontline therapy with a FLT3i	56	0
CCT + FLT3i	33 (59%)	0
LIT + FLT3i	22 (39%)	0
Single-agent FLT3i	1 (2%)	0
Number of therapies prior to first FLT3i exposure	0	2
Total FLT3i exposures (events) in salvage	40	301
CCT + FLT3i	9 (22%)	43 (14%)
LIT + FLT3i	10 (25%)	113 (38%)
Single-agent FLT3i	21 (53%)	145 (48%)
Total sequential FLT3i exposure (sequential events)	40	118

Karyotype and mutations are reported from the bone marrow prior to the first FLT3i exposure

FLT3i, FLT3 inhibitor; LIT, low-intensity chemotherapy; CCT, intensive cytotoxic chemotherapy; sAML, secondary acute myeloid leukemia

### Cohort 1

#### CRc rates by salvage status and number of prior FLT3i exposures

Fifty-six patients received FLT3i’s as part of their frontline treatment: 33 with CCT, 22 with LIT, and one single-agent quizartinib. Patient characteristics are in Table 1. The CRc was 79% (26/33) for frontline CCT with FLT3i’s: 23 of 33 with sorafenib, 8 with midostaurin, 1 with gilteritinib, and 1 with quizartinib (Table 2). The CRc was 77% (17/22) for frontline LIT with FLT3i’s: 13 of 22 received quizartinib with LIT (HMA or LDAC) with a CRc of 85%, 6, 2, and 1 received sorafenib, gilteritinib, and midostaurin-based LITs with CRc rates of 50%, 100%, and 100%, respectively. Only one treatment naïve patient received single-agent quizartinib with no response.

**Table 2:** Response rates by FLT3i exposure sequence

Therapy by cohort	N	First FLT3i	Second FLT3i	Third/fourth FLT3i	*P* value*
		Number of responders/total (CRc rate)			
Cohort 1	96	43/56 (77%)	10/32 (31%)	2/8 (25%)	–
Single-agent FLT3i	22	0/1 (0%)	2/15 (13%)	2/6 (33%)	–
LIT + FLT3i	32	17/22 (77%)	4/9 (44%)	0/1 (0%)	–
CCT + FLT3i	42	26/33 (79%)	4/8 (50%)	0/1 (0%)	–
Cohort 2	301	82/183 (45%)	19/89 (21%)	3/29 (10%)	< 0.001
Single-agent FLT3i	145	28/82 (34%)	7/47 (14%)	1/16 (6%)	0.010
LIT + FLT3i	113	40/74 (54%)	10/32 (31%)	2/7 (28%)	0.061
CCT + FLT3i	43	14/27 (52%)	2/10 (20%)	0/6 (0%)	0.026

FLT3i, FLT3 inhibitor; CRc, composite CR rate; N, number; LIT, low-intensity chemotherapy; CCT, intensive cytotoxic chemotherapy

^*^P values added for cohort 2 patients only, as numbers of patients may be too small to make meaningful comparisons in cohort 1

Among 56 patients who received a frontline FLT3i, 32 eventually relapsed or were refractory. All 32 received a second sequential FLT3i, and 8 went on to receive a third sequential FLT3i exposure for a total of 40 sequential FLT3i-based therapies in cohort 1 (Table 2).

The CRc rate in the 32 s FLT3i exposures was 31%. CRc rates with CCT (*n* = 8), LIT (*n* = 9), and single-agent (*n* = 15) FLT3i-based therapies were 50%, 44%, and 13% (*P* = 0.118), respectively, suggesting a trend for benefit with combinatorial approaches in patients failing a frontline FLT3i-based therapy (Table 2). Eight of the 32 patients went on to receive a third FLT3i exposure with CRc in 2 of 8: two received a CCT- and LIT-based combination, respectively, and did not respond, while 6 received single-agent FLT3i therapies on available clinical trials including 2 who received quizartinib with CRc in both, and 4 others (2 crenolanib, 1 AP24534, 1 E6201) with no responses (Additional file 1: Table S4B). No patient received a fourth FLT3i-based therapy in cohort 1.

#### Overall survival and event-free survival

**Fig. 2:**
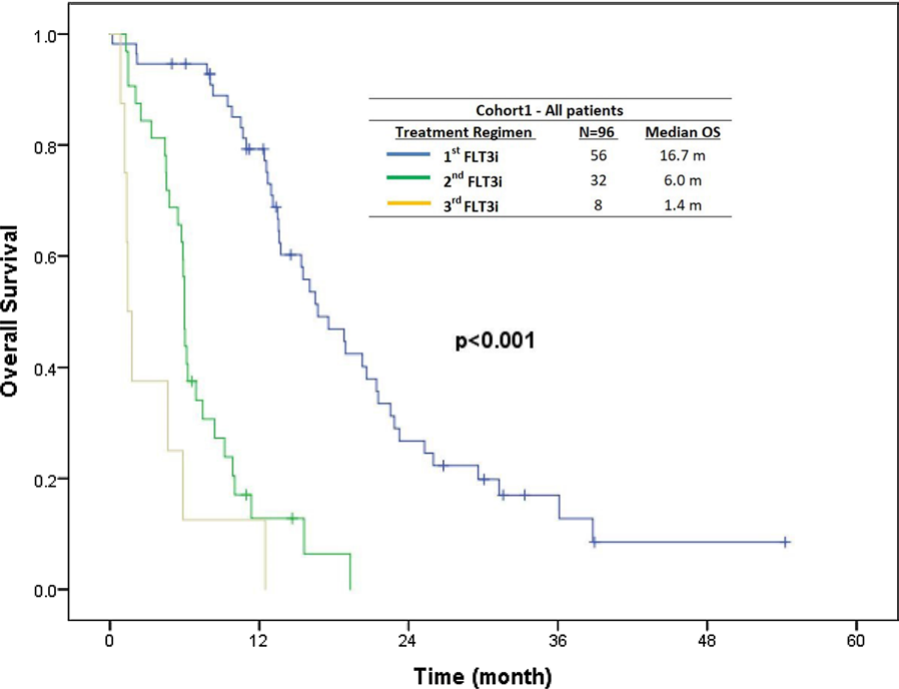
The median OS of patients in cohort 1 (frontline cohort) by FLT3i exposure sequence

The median OS was 16.7 in the frontline (first FLT3i exposure), 6.0, and 1.4 months in second and third FLT3i exposure, respectively, in cohort 1 (*P* < 0.001) (Fig. 2a). The median OS with LIT-based, CCT-based, and single-agent FLT3i in the 32 s FLT3i exposures was 5.8 months, 15.6 months, and 6.0 months, respectively (*P* < 0.001).

#### Outcomes in patients harboring FLT3 TKD mutations

In cohort 1, 41 (80%), 4 (12%), and 6 (8%) patients had ITD, D835 TKD, and ITD/D835 TKD mutations, respectively. (An additional 5 patients with no ITD versus TKD specification were excluded for this analysis.) Of 10 patients harboring D835 TKD mutations, 7 achieved CRc (70%): 4 received sorafenib (3 achieved CRc), 3 quizartinib (2 achieved CRc), and 3 midostaurin (2 achieved CRc) as induction FLT3i. All received respective FLT3i’s in combination with CCT, except 1 patient who was treated with single-agent quizartinib and did not respond. Four patients with TKD D835 mutations received a second FLT3i-based therapy (none in third FLT3i group) with CRc in 1 (25%).

### Cohort 2

#### CRc rates by salvage status and number of prior FLT3i exposures

A total of 183 patients with *FLT3*-ITD-mutated R/R AML received a FLT3i-based treatment for the first time in a salvage setting, of whom 89 received a second, 25 a third, and only 4 a fourth sequential FLT3i-based treatment (Table 2). Patient characteristics are in Table 1.

Overall, 301 (183 first FLT3i exposure in salvage, 118 s/third/fourth sequential FLT3i exposure in salvage) FLT3-inhibitor-based treatment exposures were identified in cohort 2 (schema in Fig. 1). All 301 exposures were included in this analysis: 145 single-agent FLT3i, 113 LIT with FLT3i, and 43 CCT with FLT3i. Overall (*n* = 301), CRc rates were 45%, 21%, and 10% with the first, second, and third/fourth FLT3i-based therapy exposures in the salvage AML setting, respectively (P < 0.001) (Table 2). In patients treated with single-agent FLT3i’s (*n* = 145), the CRc rates declined from 34 to 14% to 6% with first, second, and third/fourth FLT3i exposures (*P* = 0.01) (Table 2). In patients treated with LIT with FLT3i’s (*n* = 113), the CRc rates declined from 54 to 31% to 28% with the first, second, and third/fourth FLT3i exposures (*P* = 0.061). In patients treated with CCT with FLT3i (*n* = 43), the CRc rates declined from 52 to 20% to 0% with the first, second, and third/fourth FLT3i exposures (*P* = 0.026).

In the first FLT3i exposure (*n* = 183) in salvage patients, the median number of prior treatments for AML was 2 (range, 1—7); however, none included a prior FLT3i as per definition of this cohort. CRc rates with single-agent FLT3i, LIT with FLT3i, and CCT with FLT3i were 34%, 54%, and 52%, respectively (*P* = 0.032) (Table 2).

In the second sequential FLT3i exposure (*n* = 89) in salvage patients, the median number of prior treatments for AML was 3 (range, 1—8), including a median of one prior FLT3i-based treatment. CRc rates with single-agent FLT3i, LIT with FLT3i, and CCT with FLT3i were 14%, 31% and 20%, respectively (*P* = 0.218) (Table 2).

Only 25 patients received a third sequential FLT3i-based treatment in salvage. The median number of prior treatments for AML was 4 (range, 3—10), including a median of two prior FLT3i-based treatments. CRc rates with single-agent FLT3i, LIT with FLT3i, and CCT with FLT3i were 7%, 28%, and 0%, respectively (*P* = 0.255) (Table 2).

Only 4 patients received a fourth FLT3i-based treatment, 3 with single-agent FLT3i, and 1 in combination with CCT with no responses.

#### CRc rates by individual FLT3i-based therapies in cohort 2

Analyzing the data by the specific FLT3i’s used, 301 independent FLT3i-based exposures were captured in cohort 2, excluding any duplication of the same FLT3i usage in any patient (Additional file 1: Table S4A). Based on the clinical trials at our institution in this time frame, the most frequently used FLT3i’s in these 301 exposures were quizartinib [105 (35%) of all exposures] and sorafenib [89 (30%) of all exposures] (details of specific FLT3i used in Additional file 1: Table S4A).

**Fig. 3:**
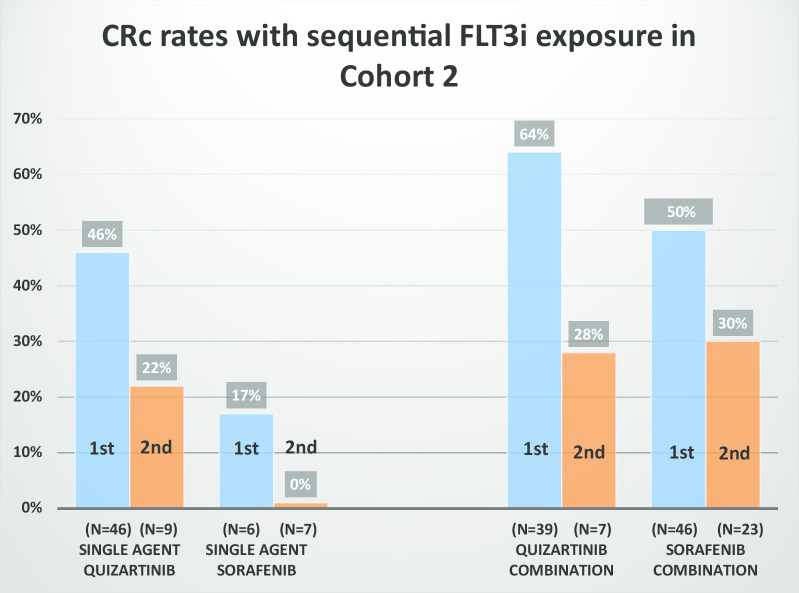
First and second FLT3i exposure in cohort 2 (relapsed/refractory) as single agent and in combinations with cytotoxic chemotherapy or low-intensity treatment

The CRc rate with single-agent quizartinib as the first FLT3i in salvage AML (*n* = 46) was 46% (Fig. 3), consistent with published phase II/III CRc rates with single-agent quizartinib in predominantly non-FLT3i-exposed patients [7, 9, 13]. The CRc rate with quizartinib-based combinations as the first FLT3i exposure in salvage AML (*n* = 39) was 64% (Fig. 3).

In the first (*n* = 46), second (*n* = 9), and third (*n* = 2) FLT3i exposures in salvage AML, CRc rates with single-agent quizartinib progressively declined: 46%, 22%, and 0% (Fig. 3), respectively, consistent with what has previously been presented for quizartinib [17], although numbers are small in later sequential therapies. The LIT combinations did better than single-agent quizartinib in both the first (*n* = 39 of the 46) and second (*n* = 7 of the 9) FLT3i exposures, with CRc rates of 64% and 28%, respectively (Fig. 3). Only 2 patients received quizartinib with LIT in the third FLT3i exposure with one response.

Quizartinib is a potent FLT3i. We noted that switching to another FLT3i after failing quizartinib-based therapies as the first FLT3i (*n* = 30) exposure in salvage AML produced a CRc rate of only 20% (*n* = 6, 5 in combination and 1 as single agent) (Additional file 1: Table S4B).

The second most common FLT3i used in salvage AML (cohort 2) was sorafenib [89 (30%) of all exposures] (Additional file 1: Table S4A). The majority (85%) of sorafenib therapies were administered in combination. CRc rates with sorafenib-based combinations in first (*n* = 46) and second (*n* = 23) FLT3i exposure settings in salvage AML were 50% and 30%, respectively (Fig. 3), similar to previously published [18], compared with CRc rates of 17% and 0% with single-agent sorafenib in the first (*n* = 6) and second (*n* = 7) FLT3i exposure settings in salvage AML. Only 5 patients received sorafenib in combination with LIT in the third FLT3i exposure setting with 1 (20%) CRc.

Switching to another FLT3i after failing sorafenib-based therapies as the first FLT3i (*n* = 35) in salvage was associated with a CRc rate of 17% (Additional file 1: Table S4C). Quizartinib-based therapies maintained a degree of efficacy with a CRc rate of 25% as second FLT3i-based therapies post-sorafenib failure (*n* = 12) (Additional file 1: Table S4C).

Gilteritinib was used in 13 patients in salvage in this time frame (Additional file 1: Table S4A). Eleven patients received single-agent gilteritinib, with 3 and 8, receiving it in the first and second/third FLT3i exposure settings, with respective CRc rates of 67% and 50%. Two patients received gilteritinib with LIT; 1 achieved CRc. No patient received a FLT3i-based therapy after failing gilteritinib, so this could not be analyzed.

Midostaurin was always used in combination (*n* = 10), with 50% (3 of 6) and 25% (1 of 4) CRc’s in the first and second FLT3i exposures in salvage, respectively (Additional file 1: Table S4A). Although the sample is small (*n* = 7), post-midostaurin CRc rates to second-generation FLT3i-based therapies were 29% (2 of 7 responses: to quizartinib- and crenolanib-based therapies) suggesting the potential activity of second-generation FLT3i’s post-midostaurin (Additional file 1: Table S4D).

Other phase 1 FLT3i-based therapies were given to 18 patients in various salvage settings, including 6 patients who received E6201, 5 FLX925, 2 KW-2449, and 1 each SAR103168, AP24534, CEP-701, TG02, and SEL25. All were used as single agent except CEP-701 (in combination with CCT). The median number of prior FLT3i exposures prior to receiving these phase 1 FLT3i’s was 4 (range 1–8). No patients achieved a CRc in this group (Additional file 1: Table S4A).

#### Overall survival and event-free survival in cohort 2

**Fig. 4:**
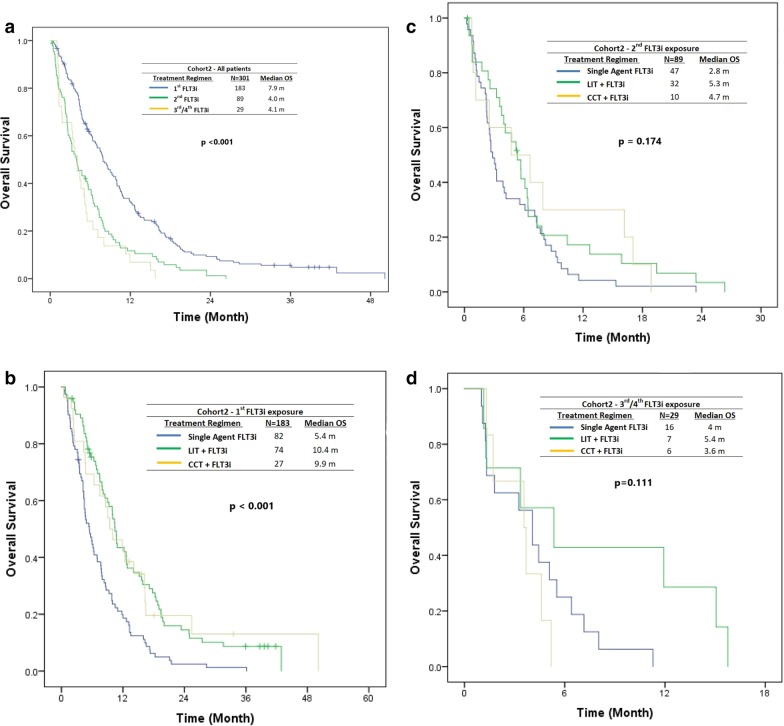
The median OS in cohort 2 (relapsed/refractory) by sequence of FLT3i exposure (**a**), and treatment modality (**b**–**d**) (single-agent FLT3i vs. combination)

In cohort 2 (R/R AML), the median OS was 7.9 months, 4.0 months, and 4.1 months with the first (*n* = 183), second (*n* = 89), and third/fourth (*n* = 29) FLT3i exposure, respectively (*P* < 0.001) (Fig. 4a). Median OS with the first FLT3i-based therapy exposure in salvage AML was 5.4, 10.4, and 9.9 months with single-agent, LIT, and CCT FLT3i-based therapies, respectively (*P* < 0.001) (Fig. 4b). Median OS with the second FLT3i-based therapy exposure in salvage AML was 2.8, 5.3, and 4.7 months, with single-agent, LIT, and CCT FLT3i-based therapies, respectively (*P* = 0.174) (Fig. 4c), implying 2.8 months compared with 5.4 months (*P* = 0.06) for single-agent versus combination therapies of FLT3i’s. Median OS with the third FLT3i-based therapy exposure in salvage AML was 4.0, 5.4, and 3.6 months with single-agent, LIT, and CCT FLT3i-based therapies, respectively (*P* = 0.111) (Fig. 4d).

### Outcomes in patients harboring FLT3 TKD mutations

In cohort 2, of 183 patients, 107 (84%), 15 (12%), and 6 (4%) patients had ITD alone, D835 TKD alone, and ITD + D835 TKD mutations, respectively. Additionally, 55 FLT3-mutated patients with no ITD versus TKD specification were excluded for this analysis. Of the 21 patients harboring D835 TKD mutations, 6 achieved CRc (30%): 14 received crenolanib (4 achieved CRc), 3 quizartinib (1 CRc), 2 gilteritinib (1 CRc), and 2 sorafenib (no CRc) as first FLT3i. Of the 6 responses, 2 were with CCT and 4 were single-agent FLT3i. Fourteen and 6 patients harboring a TKD D835 mutations received a second and third/fourth FLT3i-based therapy with CRc in 3 (21%) and 2 (33%), respectively.

#### Impact of MRD at CRc by MFC and FLT3 PCR in cohort 2 patients

**Fig. 5:**
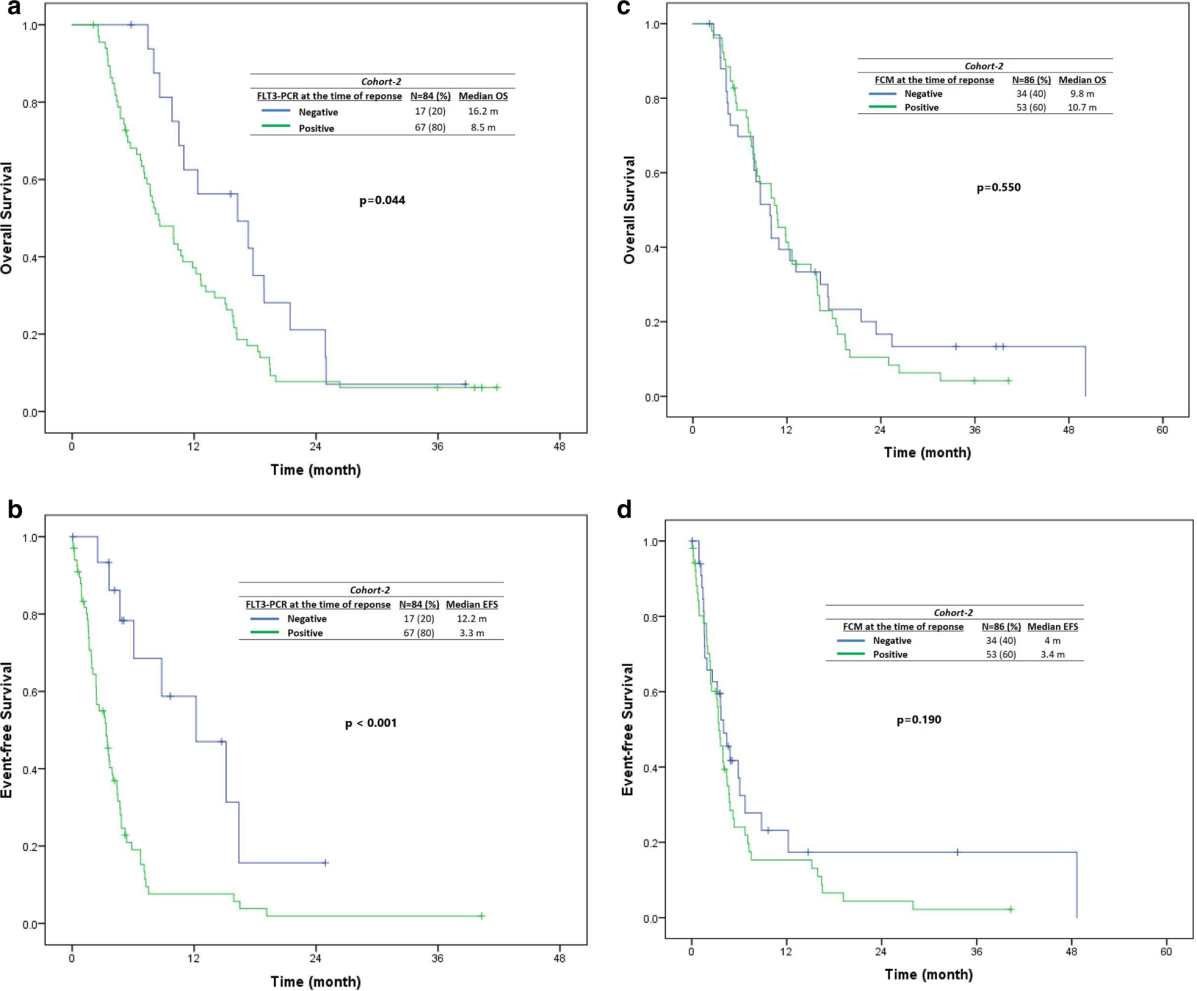
Impact of minimal residual disease status on survival by FLT3-PCR [A-B] and multiparametric flow cytometry [C-D]) in cohort 2 (relapsed/refractory) patients who achieved a CRc. In cohort 2 (relapsed/refractory), among the responders with FLT-ITD PCR checked at the time of CRc, patients who achieved minimal residual disease (MRD) negativity by FLT3 RT-PCR had improved OS and EFS (**a**, **b**). In contrast, there was no significant impact of achieving MRD negativity by multiparametric flow cytometry (C and D) on OS and EFS in R/R FLT3-mutated AML treated with FLT3i-based therapies

In R/R AML group (cohort 2), 104 of 301 achieved CRc, and 84 of 104 (80%) of patients who achieved CRc had serial *FLT3*-ITD/TKD PCR checked on bone marrow at baseline and at CRc. (Median time to MRD assessment from documentation of the first CRc response was 32 days [range, 13–305 days].) Seventeen of 84 (20%) CRc patients achieved minimal residual disease (MRD) negativity by *FLT3*-PCR. Patients who achieved MRD negativity by *FLT3*-PCR had improved OS (16.3 versus 8.5 months, *P* = 0.04) and event-free survival (censored for transplant) (12.2 versus 3.3 months, *P* < 0.001) (Fig. 5a, b). The FLT3-PCR negativity rates are in first FLT3i exposure (16/65; 25%), the second FLT3i exposure (1/16, 6%), and third/fourth FLT3i exposure settings (0/3, 0%).

In R/R AML group (cohort 2), 86 of 104 (82%) had MRD by MFC checked on bone marrows serially at baseline and at CRc. Thirty-four of 86 (39%) achieved MRD-negative status by MFC using the described technique. As opposed to *FLT3*-PCR clearance, achievement of MRD negativity by MFC at CRc was not associated with a significant impact on OS (9.8 vs 10.7 months, *P* = 0.55) nor EFS (censored for transplant) (4 vs 3.4 months, *P* = 0.19) (Fig. 5c, d). The MFC negativity rates are in first FLT3i exposure (33/67; 49%), the second FLT3i exposure (1/16, 6%), and third/fourth FLT3i exposure settings (0/3, 0%).

We identified a correlation between MFC and PCR results. Overall, 75 patients who achieved CRc had MRD assessment performed with both MFC and PCR at response. Of 17 PCR-negative patients, 13 (76%) were also MFC negative, and of 58 PCR-positive patients, 42 (72%) were positive by MFC (*P* = 0.001) (Additional file 1: Table S5).

## Discussion

In our analysis, CRc rates and median OS dropped with sequential FLT3i exposure. Frontline FLT3i (midostaurin) added to induction is the recommended and widely followed approach for newly diagnosed FLT3 (ITD or D835)-mutated AML based on the phase III RATIFY results [3, 19]. Our data suggest that expected CRc rates with sequential FLT3i-based therapies in contemporaneously treated patients will be lower (25–31%), than the published CRc rates of 45–55%, reported in phase II/III trials of second-generation FLT3i’s (gilteritinib [5, 6] and quizartinib [7, 9, 13]), as the majority (85—90%) of patients on those trials (conducted prior to midostaurin approval) were prior FLT3i naïve. Although lower, the CRc rates with sequential use of second-generation FLT3i’s are still clinically meaningful and appeared to be higher with combinatorial approaches. These findings have direct practical implications for treating leukemia physicians.

Our findings may also be important for ongoing FLT3i clinical research. The CRc rate in combined cohorts 1 and 2 was 24% and 13% in the second (*n* = 29/121) and third/fourth (*n* = 5/37) sequential FLT3i exposure setting (single agent and combined), respectively. For single-agent FLT3i’s, the CRc rates in combined cohorts 1 and 2 were lower at 15% and 14% in the second (*n* = 9/62) and third/fourth (*n* = 3/22) exposure setting, respectively (Additional file 1: Table S3, S4A). For CCT and LIT FLT3i combinations, the CRc in combined cohorts 1 and 2 was 47% and 28% in the second (*n* = 8/17, 12/42) and 0% and 15% third/fourth (*n* = 0/2, 2/13) exposure setting, respectively (Table 2). These data provide a hitherto not available benchmark for CRc rates and OS in the contemporary post-RATIFY era for evaluating second, and in the even more contemporary post-ADMIRAL era for evaluating third FLT3i-based therapy exposures. Perl A et al. recently reported a CRc rate of 88% in R/R FLT3-mutated patients treated with gilteritinib and venetoclax in a phase 1B study. The CRc rate was 85% in patients with R/R *FLT3*-mutated AML previously exposed to FLT3i. While these data are encouraging on their own, the 85% CRc rate gains more significance when we consider that the benchmark expectations for CRc in such prior TKI-exposed patients based on this analysis would in fact be 20–30% and not 45–55% as reported in the ADMIRAL and QuANTUM-R trials. Establishing appropriate benchmarks for second and third FLT3i exposure based on the current treatment paradigm will allow us to critically analyze emerging data from ongoing trials, thereby avoiding false-negative adjudications on trials or discarding drugs/combinations that may in fact be showing encouraging activity when correctly analyzed using contemporary benchmarks.

It is important to note the difference in single-agent versus combinations. The CRc rates and median OS with all sequential FLT3i exposures (excluding the first FLT3i exposure) with single-agent, LIT, and CCT FLT3i-based therapies across cohorts 1 and 2 were 14% (12/84), 33% (16/49), and 24% (6/25) and 4.4 m, 7.5 m, and 8.6 m, respectively. While this analysis incorporates a number of different FLT3i-based single-agent and combinatorial approaches across multiple trials, which could introduce a number of potential biases, the general theme that emerges across both cohorts 1 and 2 is that combinatorial approaches may be associated with improved CRc rates and OS. In cohort 2, CRc rates with LIT and CCT were similar, 54% versus 52%, respectively, in the first FLT3i exposure setting (Table 2). Although patient numbers are smaller, with second or third/fourth FLT3i exposures, CRc rates appeared to be better with LIT with FLT3i versus CCT with FLT3i regimens, 31% versus 20% and 28% versus 0%, respectively. LIT with FLT3i appears to be feasible and effective and may be a better tolerated sequential FLT3i option compared with CCT with FLT3i, particularly in patient relapsing after a prior intensive regimen. However, randomized studies of LIT-based versus CCT-based combinations or larger single-arm studies using more homogenous LIT or CCT backbones with the same FLT3i added would be needed to draw more definitive conclusions.

Based on the clinical trials conducted at our center in the last 13–14 years (period of this analysis), the four FLT3i’s commonly used in salvage, either as first FLT3i exposure or as sequential FLT3i exposure in our analysis, were sorafenib, crenolanib, quizartinib, and gilteritinib. Ravandi et al. documented a CR/CRi rate of 42% in R/R FLT3-ITD-mutated patients with azacitidine with sorafenib [18]. Among prior FLT3i-exposed patients in their study, 3 (33%) achieved CR/CRi with azacitidine and sorafenib. Similarly, we noted a CRc rate of 50% in 46 FLT3-mutated R/R patients treated with sorafenib-based combinations (CCT = 20, LIT = 26) as first FLT3i exposure, likely with some overlap for LIT patients from the Ravandi et al. patients. Among 29 patients exposed to one prior FLT3i, 30% (7 of 23) achieved a CRc with sorafenib-based combinations. This suggests that sorafenib-based combinations remain a reasonable option in patients with R/R FLT3 AML who have failed a prior FLT3i-based therapy as outlined in the NCCN guidelines [20].

Quizartinib has published single-agent CRc rate of 48% in the phase III QuANTUM-R study. 96% of the patients in that study had no prior FLT3 TKI exposure. Similarly, in our analysis, the CRc rate was 46% in 46 patients who received single-agent quizartinib as the first FLT3i in salvage. CRc rates with single-agent quizartinib dropped to 22% in 9 patients who received quizartinib after exposure to one prior FLT3i. These findings are consistent with a post hoc analysis of two phase II trials of quizartinib monotherapy (NCT01565668 and NCT00989261), wherein single-agent quizartinib CRc rates were 48–53% in prior FLT3 TKI naïve compared with 33–36% in prior FLT3 TKI-exposed patients [17]. LIT combinations with quizartinib showed high efficacy with CRc rates of 64% and 33% and median OS of 10.7 and 6.1 in prior FLT3i naïve and prior FLT3i-exposed R/R patients, respectively, consistent with previous data [21].

Gilteritinib was only used as a single-agent and in R/R setting (12 patients) during the time period analyzed. CRc rates were 67% and 38%, in 3 FLT3 TKI naïve and 8 patients exposed to one prior FLT3 TKI, respectively. One additional patient received gilteritinib after two prior FLT3 TKIs and achieved CRi. These numbers are small; however, they are similar to CRc rates of 41–54% with gilteritinib monotherapy in predominantly FLT3 TKI naïve patients in published phase II/III studies, and CRc rate of 26% with gilteritinib monotherapy in a subset of 57 patients who had received a prior FLT3 TKI in the gilteritinib phase II CHRYSALIS study.

## Conclusion

Second-generation FLT3i’s quizartinib and gilteritinib have high activity as single-agents in patients with R/R FLT3 AML who are FLT3 TKI naïve (46–67% CRc) or exposed to one prior FLT3 TKI (22–38% CRc). FLT3i combinations had higher response rates and improved OS compared with single-agent FLT3i’s in similar FLT3i exposure settings. Numerous trials evaluating combinations of FLT3i’s with induction chemotherapy, hypomethylating agents, venetoclax, and triplets of hypomethylating agents with venetoclax and FLT3i’s are ongoing (NCT03661307, NCT04140487, NCT03735875) and will hopefully improve response rates and survival. These should be strongly considered for R/R FLT3-mutated AML patients, especially for patients who have failed a prior FLT3i-based therapy.

## Supplementary information

**Additional file 1**. Manuscript Supplementary Information.

## Data Availability

The datasets used and/or analyzed during the current study are available from the corresponding author on reasonable request.

## Abbreviations

FLT3i: FLT3 inhibitor
AML: Acute myeloid leukemia
R/R: Relapsed/refractory
CRc: Composite complete remission rate
ITD: Internal tandem duplication
IRB: Institutional Review Board
UT/MDACC: The University of Texas MD Anderson Cancer Center
CCT: Intensive cytotoxic chemotherapy
LIT: Low-intensity therapy
EFS: Event-free survival
OS: Overall survival
MFC: Multiparametric flow cytometry
HMA: Hypomethylating agent
LDAC: Low-dose cytarabine
MRD: Minimal residual disease
PCR: Polymerase chain reaction
TKI: Tyrosine kinase inhibitor
NCCN: National comprehensive cancer network
N: Number
