# Correction to: Outcomes with sequential FLT3-inhibitor-based therapies in patients with AML

**DOI:** 10.1186/s13045-020-01023-9

**Published:** 2021-02-23

**Authors:** Musa Yilmaz, Mansour Alfayez, Courtney D. DiNardo, Gautam Borthakur, Tapan M. Kadia, Marina Y. Konopleva, Sanam Loghavi, Rashmi Kanagal-Shamanna, Keyur P. Patel, Elias J. Jabbour, Guillermo Garcia‑Manero, Naveen Pemmaraju, Sherry A. Pierce, Issa Ghayas, Nicholas J. Short, Guillermo Montalban-Bravo, Koichi Takahashi, Rita Assi, Ahmad S. Alotaibi, Maro Ohanian, Michael Andreeff, Jorge E. Cortes, Hagop M. Kantarjian, Farhad Ravandi, Naval G. Daver

**Affiliations:** 1 Department of Leukemia, The University of Texas MD Anderson Cancer Center, 1515 Holcombe Blvd, Unit FC4.2012, Houston, TX 77030 USA; 2 Department of Hematopathology, The University of Texas MD Anderson Cancer Center, Houston, TX USA

## Correction to: J Hematol Oncol (2020) 13:132 10.1186/s13045-020-00964-5

The original article [18] mistakenly contains duplicate references. The correct reference list can be viewed in this Correction article.
