# Supplementary material for: Outcomes with sequential FLT3-inhibitor-based therapies in patients with AML
Source: J Hematol Oncol. 2020 Oct 8;13:132. doi: 10.1186/s13045-020-00964-5 (PMC7542942; doi:10.1186/s13045-020-00964-5)
Supplement: Supplementary file 1 — Additional file 1. Manuscript Supplementary Information. [file 13045_2020_964_MOESM1_ESM.docx]

Supplementary

**INDEX:**

Page: 2 Supplement Table 1: List of clinical trials included in this analysis.

Page: 4 Supplement Table 2: Molecular and Multicolor Flow Cytometry Assessments

Page: 8 Supplement Table 3: Detailed cohort 1 responses.

Page: 9 Supplement Table 4 A&D: Detailed cohort 2 responses.

Page 11 Supplementary Table 5. Correlation of Assessments by MFC and PCR at the time of response

Page 11 Supplementary table 6. Response rates based on FLT3-ITD, DMNT3A and NPM1 status

Page 11 Incidence of pretherapy FLT3-D835 mutation with sequential FLT3i exposure (in cohort-2)

Page 11 FLT3 allelic frequency in sequential FLT3-inhibitor settings in Cohort 2

Page 12 Supplementary Figure 1 (A-C) Overall Survival patients with FLT3-ITD, DNMT3A and NPM1 co-mutated patients.

**Supplementary table 1: List of clinical trials included in this analysis.**

| **Study Name** | **# of pts** | **MDA protocol** | **NCT#** |
| --- | --- | --- | --- |
| Study of CEP-701 (Lestaurtinib) in Patients With Acute Myeloid Leukemia (AML) | 1 | 2003-0719 | NCT00079482 |
| Sorafenib in Treating Patients With Refractory or Relapsed Acute Leukemia, Myelodysplastic Syndromes, or Blastic Phase Chronic Myelogenous Leukemia | 5 | 2004-0702 | NCT00217646 |
| An Ascending Dose Study of KW-2449 in Acute Leukemias, Myelodysplastic Syndromes, and Chronic Myelogenous Leukemia | 2 | 2006-0275 | NCT00346632 |
| A Phase I Study of AC220 in Patients With Relapsed/Refractory Acute Myeloid Leukemia Regardless of FLT3 Status | 28 | 2006-0850 | NCT00462761 |
| Phase I-II Study of Idarubicin, Cytarabine, and Sorafenib (BAY43-9006) | 7 | 2006-0977 | NCT00542971 |
| Safety Study of AP24534 to Treat Chronic Myelogenous Leukemia (CML) and Other Hematological Malignancies | 2 | 2008-0046 | NCT00660920 |
| Granulocyte-colony Stimulating Factor (G-CSF) and Plerixafor Plus Sorafenib for Acute Myelogenous Leukemia (AML) With FLT3 Mutations | 9 | 2008-0501 | NCT00943943 |
| Dose Escalation, Safety and Pharmacokinetic Study of SAR103168 in Patients Refractory/ Relapsed Acute Leukemias or High-risk Myelodysplastic Syndromes | 1 | 2009-0196 | NCT00981240 |
| Efficacy Study for AC220 to Treat Acute Myeloid Leukemia (AML) (ACE) | 30 | 2009-0560 | NCT00989261 |
| Phase 1 Study of TG02 Citrate in Patients With Advanced Hematological Malignancies (TG02-101) | 1 | 2010-0244 | NCT01204164 |
| PKC412 and 5-Azacytidine | 9 | 2010-0374 | NCT01202877 |
| Sorafenib and 5-Azacitidine in Acute Leukemia + Myelodysplastic Syndrome | 13 | 2010-0511 | NCT01254890 |
| A Study to Assess AC220 Given in Combination With Induction and Consolidation Therapy in Newly Diagnosed Acute Myeloid Leukemia (AML) | 1 | 2011-0041 | NCT01390337 |
| Open Label Study to Evaluate Safety and Efficacy of 2 Doses of Quizartinib in Patients With Relapsed or Refractory Acute Myeloid Leukemia | 6 | 2012-0054 | NCT01565668 |
| A Phase II Study of Crenolanib in Relapsed/Refractory Acute Myeloid Leukemia Patients With FLT3 Activating Mutations | 56 | 2012-0569 | NCT01657682 |
| Cladribine Plus Idarubicin Plus Cytarabine (ARAC) in Patients With Acute Myeloid Leukemia (AML), High Risk Myelodysplastic Syndrome (HR MDS) or Myeloid Blast Phase of Chronic Myeloid Leukemia (CML) | 5 | 2012-0648 | NCT02115295 |
| Quizartinib With Azacitidine or Cytarabine in Treating Participants With Relapsed or Refractory Acute Myeloid Leukemia or Myelodysplastic Syndrome | 63 | 2012-1047 | NCT01892371 |
| Dose Escalation Study Investigating the Safety, Tolerability, Pharmacokinetics, Pharmacodynamics of ASP2215 in Patients With Relapsed or Refractory Acute Myeloid Leukemia | 10 | 2013-0672 | NCT02014558 |
| Sorafenib Plus 5-Azacitidine Initial Therapy of Patients With Acute Myeloid Leukemia (AML) and High Risk Myelodysplastic Syndrome (MS) With FLT3-ITD Mutation | 2 | 2014-0076 | NCT02196857 |
| A Study of E6201 for the Treatment of Advanced Hematologic Malignancies With FLT3 and/or Ras Mutations | 7 | 2014-0777 | NCT02418000 |
| Phase I/II, Study of Selective Inhibitor of Nuclear Export (SINE) Selinexor (KPT-330) + Sorafenib in Acute Myeloid Leukemia | 3 | 2014-0975 | NCT02530476 |
| First-in-Human Study of FLX925 in Subjects With Relapsed or Refractory Acute Myeloid Leukemia | 5 | 2014-1000 | NCT02335814 |
| Study of Crenolanib Combined With Chemotherapy in FLT3-mutated Acute Myeloid Leukemia Patients | 22 | 2015-0207 | NCT02400281 |
| SEL24/MEN1703 in Patients With Acute Myeloid Leukemia | 1 | 2016-0713 | NCT03008187 |
| Off protocol | 71 | NA | NA |

**Supplementary table 2: Coverage by gene and codon(s) tested**

**Table 2A. 81 gene panel**

**Gene Exons (codons) tested**

ANKRD26 (NM_014915) 1 (1-6)

ASXL1 (NM_015338) 11-12 (362-1442), 12 (1450-1542)

ASXL2 (NM_018263) 11-12 (381-1436)

BCOR (NM_017745) 2-4 (1-511), 4-15 (515-1644), 15 (1663-1722)

BCORL1 (NM_021946) 1-6 (1-1261), 6 (1292-1323), 6-12 (1326-1700), 12 (1706-1712)

BRAF (NM_004333) 11 (439-478), 15 (581-620)

BRINP3 (NM_199051) 2-8 (1-767)

CALR (NM_004343) 9 (352-418)

CBL (NM_005188) 7-9 (336-477)

CBLB (NM_170662) 7-9 (282-397), 10 (402-469)

CBLC (NM_012116) 7-9 (336-454), 10 (465-475)

CEBPA (NM_004364) 1 (1-96), 1 (249-358), 1 (215-244), 1 (128-175), 1 (178-201)

CREBBP (NM_004380) 1-8 (1-608), 9-31 (615-1943), 31 (1950-2443)

CSF3R (NM_156039) 14 (575-622), 17 (681-864)

CUX1 (NM_181552) 2-6 (11-172), 6-9 (174-241), 10-14 (248-408)

DDX41 (NM_016222) 1-17 (1-623)

DNMT3A (NM_022552) 8-22 (286-862), 23 (866-913)

EED (NM_003797) 1-2 (1-69), 2-8 (71-287), 9-12 (289-442)

ELANE (NM_001972) 1-2 (1-48), 2 (69-75), 3-5 (102-268)

ETNK1 (NM_018638) 3 (228-275)

ETV6 (NM_001987) 1-8 (1-453)

EZH2 (NM_004456) 2-5 (1-158), 5-6 (160-205), 7 (209-217), 8-19 (243-732), 20 (752)

FBXW7 (NM_033632) 9-12 (413-708)

FLT3 (NM_004119) 11-20 (437-847)

GATA1 (NM_002049) 2-3 (1-84)

GATA2 (NM_032638) 2-5 (1-377), 5-6 (379-481)

GFI1 (NM_005263) 2 (2-39)

GNAS (NM_000516) 8 (200-202), 11 (315-324)

HNRNPK (NM_002140) 3-17 (1-465)

HRAS (NM_005343) 2-3 (1-70), 3-4 (74-150)

IDH1 (NM_005896) 4 (132-133)

IDH2 (NM_002168) 4 (125-178)

IKZF1 (NM_006060) 2-8 (1-443), 8 (445-518)

IL2RG (NM_000206) 1-8 (1-370)

IL7R (NM_002185) 5-7 (180-292)

JAK1 (NM_002227) 3-22 (3-1023), 22-24 (1026-1123)

JAK2 (NM_004972) 10 (405-442), 12-14 (505-622), 16 (665-711), 18 (762-803)

JAK3 (NM_000215) 2-23 (1-1069)

KDM6A (NM_021140) 1-19 (1-971), 19-21 (980-1070), 22-29 (1080-1402)

KIT (NM_000222) 8-9 (411-514), 11 (550-592), 17 (788-828)

KMT2A (NM_005933) 2 (145-168), 3-4 (176-1075), 4 (1081-1112), 5 (1117-1184), 6 (1190-1212), 7 (1224-1325), 8-10 (1338-1440), 11-13 (1445-1560), 14-15 (1566-1665), 27 (2186-2195), 27 (2201-2355), 27 (2373-3215), 27 (3223-3324), 27 (3339-3575)

KRAS (NM_004985) 2-4 (1-150)

MAP2K1 (NM_002755) 2 (27-90), 3 (98-146)

MPL (NM_005373) 10 (490-522), 12 (552-636)

NF1 (NM_001042492) 2-5 (21-189), 6 (201-218), 8-13 (244-467), 13-17 (478-667), 18 (674-728), 18-22 (746-992), 23-24 (997-1066), 25-26 (1082-1146), 26-30 (1160-1370), 31-35 (1382-1550), 35-38 (1564-1868), 39 (1870-1884), 39-47 (1886-2322), 47-52 (2325-2555), 52-58 (2568-2840)

NOTCH1 (NM_017617) 26-28 (1529-1795), 34 (2061-2286), 34 (2290-2556), 34 (2061-2286), 34 (2290-2556)

NPM1 (NM_002520) 11 (283-295)

NRAS (NM_002524) 2-4 (1-150)

PAX5 (NM_016734) 1-10 (8-392)

PHF6 (NM_032458) 2-3 (1-78), 4-10 (81-366)

PIGA (NM_002641) 2 (1-6), 2-6 (16-485)

PML (NM_033238) 3 (201-255)

PRPF40B (NM_001031698) 2-19 (2-609), 19-20 (611-658), 20-26 (661-893)

PTEN (NM_000314) 7-8 (212-285), 8 (310-339)

PTPN11 (NM_002834) 3-4 (46-125), 7 (253-285), 12 (460-462), 12-13 (465-533)

RAD21 (NM_006265) 2-3 (1-82), 4-14 (92-632)

RARA (NM_000964) 6-7 (211-338)

RUNX1 (NM_001754) 2-9 (1-438), 9 (456-474)

SETBP1 (NM_015559) 4 (838-885)

SF1 (NM_004630) 1-13 (1-640)

SF3A1 (NM_005877) 1-9 (1-424), 9-16 (427-794)

SF3B1 (NM_012433) 13-16 (574-790)

SH2B3 (NM_005475) 2 (1-121), 2 (129-170), 2 (189-205), 2-8 (211-576)

SMC1A (NM_006306) 1-25 (1-1234)

SMC3 (NM_005445) 1 (1-5), 2-6 (19-110), 6-16 (113-504), 16-17 (507-580), 17-25 (591-975), 25-27 (979-1151), 28-29 (1159-1217)

SRSF2 (NM_003016) 1 (1-38), 1 (45-121)

STAG1 (NM_005862) 2 (1-5), 3-12 (10-392), 13-20 (402-703), 21-22 (718-738), 22-27 (740-953), 27-34 (955-1259)

STAG2 (NM_006603) 2-15 (1-512), 16-20 (521-699), 21-33 (714-1232)

STAT3 (NM_139276) 17-22 (489-715)

STAT5A (NM_003152) 3-7 (1-214), 8-9 (249-286), 9-20 (303-795)

STAT5B (NM_012448) 16 (636-693)

SUZ12 (NM_015355) 1-2 (17-107), 4-5 (129-169), 7-16 (198-740)

TERT (NM_198253) 1 (1-24), 1-2 (33-172), 2-4 (246-630), 4-16 (633-1133)

TET2 (NM_001127208) 3 (1-77), 3 (91-826), 3 (829-853), 3-11 (867-2003)

TP53 (NM_000546) 2 (1-25), 4-11 (80-394)

U2AF1 (NM_006758) 2 (15-44), 6 (117-161)

U2AF2 (NM_007279) 1-5 (1-161), 6-12 (163-473)

WT1 (NM_024426) 1 (122-216), 1 (2-59), 1 (70-104), 2-10 (216-518)

ZRSR2 (NM_005089) 1-4 (1-90), 5 (108-131), 6-9 (134-263), 9-11 (268-483)

Note. *CRLF2* (NM_022148.2) exon 6 (codons 217-256) is also targeted in this assay, and coverage for this area has been manually reviewed to be adequate. **Coverage by gene and codon(s) tested with >250x coverage.**

**Table 2B. 53 gene panel**

**Gene Exons (codons) tested**

ABL1 (NM_005157) 4-6 (243-362), 7 (395-424)

AKT1 (NM_005163) 3 (16-49)

ALK (NM_004304) 23 (1172-1175), 25 (1248-1275)

APC (NM_000038) 16 (875-918), 16 (1113-1153), 16 (1257-1575)

ATM (NM_000051) 8 (353-355), 9 (409-412), 12 (601-633), 17 (846-880), 26 (1308-1331), 34 (1678-1719), 35 (1741-1773), 36 (1792-1832), 39 (1940-1973), 50 (2441-2479), 54 (2665-2670), 55 (2694-2717), 56 (2725-2756), 59 (2889-2891), 61 (2946-2950), 63 (3007-3051)

BRAF (NM_004333) 11 (439-471), 15 (581-606)

CDH1 (NM_004360) 3 (77-117), 8 (369-379), 9 (399-439)

CDKN2A (NM_000077) 2 (51-70)

CSF1R (NM_005211) 7 (297-301), 22 (926-970)

CTNNB1 (NM_001904) 3 (12-50)

DNMT3A (NM_022552) 23 (866-913)

EGFR (NM_005228) 3 (108-142), 7 (288-297), 15 (598-627), 18-20 (708-817), 21 (857-875)

ERBB2 (NM_004448) 19 (754-769), 20 (772-818), 21 (839-883)

ERBB4 (NM_005235) 3 (98-140), 4 (153-186), 6 (208-244), 7 (248-287), 8 (295-306), 9 (333-350), 15 (579-619), 23 (907-936)

EZH2 (NM_004456) 16 (618-649)

FBXW7 (NM_033632) 5 (243-278), 8 (375-394), 9 (429-471), 10 (473-508), 11 (549-583)

FGFR1 (NM_015850) 4 (120-126), 7 (247-250)

FGFR2 (NM_000141) 7 (250-313), 9 (362-382), 12 (521-550)

FGFR3 (NM_000142) 7 (247-288), 9 (379-422), 14-15 (639-659), 18 (792-807)

FLT3 (NM_004119) 11 (437-456), 14 (569-605), 16 (648-683), 20 (807-843)

GNA11 (NM_002067) 4-5 (159-216), 6-7 (255-360)

GNAQ (NM_002072) 4-7 (159-360)

GNAS (NM_000516) 8 (200-220)

HNF1A (NM_000545) 3 (205-238), 4 (271-314)

HRAS (NM_005343) 2 (1-15), 3 (38-63)

IDH1 (NM_005896) 4 (90-132)

IDH2 (NM_002168) 4 (125-178)

JAK2 (NM_004972) 14 (615-622)

JAK3 (NM_000215) 13 (568-573), 16 (683-723)

KDR (NM_002253) 6 (220-248), 7 (267-276), 11 (471-476), 19 (872-874), 21 (946-985), 26 (1135-1146), 27 (1171-1211), 30 (1308-1357)

KIT (NM_000222) 2 (51-93), 9-11 (502-592), 13 (641-664), 14 (670-712), 15 (714-745), 17 (815-828), 18 (838-866)

KLHL6 (NM_130446) 1 (1-98)

KRAS (NM_004985) 2 (1-22), 3 (38-63), 4 (103-147)

MET (NM_001127500) 2 (168-209), 2 (375-400), 14 (1008-1028), 16 (1110-1132), 19 (1247-1284)

MLH1 (NM_000249) 12 (383-426)

MPL (NM_005373) 10 (514-522)

NOTCH1 (NM_017617) 26 (1562-1601), 27 (1673-1679)

NPM1 (NM_002520) 11 (283-295)

NRAS (NM_002524) 2 (1-18), 3 (38-62)

PDGFRA (NM_006206) 12 (552-592), 14 (659-668), 15 (673-717), 18 (823-854)

PIK3CA (NM_006218) 2 (83-118), 5 (345-353), 8 (418-445), 10 (538-555), 14 (701-729), 21 (988-1069)

PTEN (NM_000314) 1 (5-27), 3 (67-70), 6 (170-210), 7 (212-266), 8 (287-342)

PTPN11 (NM_002834) 3 (59-104), 13 (501-533)

RB1 (NM_000321) 4 (127-158), 6 (199-203), 11 (357-376), 18 (570-605), 20 (659-700), 21 (703-733), 22 (746-775)

RET (NM_020975) 10-11 (610-667), 13 (766-798), 15 (880-910), 16 (918-934)

SMAD4 (NM_005359) 3 (119-142), 5 (167-208), 6 (243-263), 8 (310-319), 9 (329-373), 10 (385-424), 11 (443-480), 12 (496-535)

SMARCB1 (NM_003073) 2 (39-78), 4 (156-167), 5 (199-210), 9 (381-386)

SMO (NM_005631) 3 (197-242), 5 (323-366), 6 (403-422), 9 (533-551), 11 (639-646)

SRC (NM_005417) 14 (530-537)

STK11 (NM_000455) 1 (36-77), 4-5 (193-211), 6 (261-288), 8 (332-370)

TP53 (NM_000546) 2 (1-12), 4 (69-112), 5-7 (126-253), 8 (267-306), 10 (332-342)

VHL (NM_000551) 1 (88-114), 2 (129-155), 3 (157-200)

XPO1 (NM_003400) 14-15 (501-575)

**Table 2C. 28 gene panel**

**Gene Exons (codons) tested**

ABL1 (NM_005157) 1-10 (1-523), 11 (560-638), 11 (661-1131)

ASXL1 (NM_015338) 2-12 (20-1542)

BRAF (NM_004333) 2-11 (47-465), 12-17 (478-709), 18 (729-767)

DNMT3A (NM_022552) 2-3 (1-59), 4-6 (89-196), 7-8 (214-322), 9-20 (339-803), 22-23 (827-913)

EGFR (NM_005228) 1-28 (1-1133), 28 (1171-1211)

EZH2 (NM_004456) 2-20 (1-752)

FLT3 (NM_004119) 2-24 (15-994)

GATA1 (NM_002049) 2-3 (1-182), 4-6 (200-336)

GATA2 (NM_032638) 2 (1-20), 3 (77-140), 3 (181-220), 3 (242-291), 4-5 (318-381), 6 (399-481)

HRAS (NM_005343) 2-4 (18-129), 5 (151-190)

IDH1 (NM_005896) 3 (1-13), 4-10 (41-415)

IDH2 (NM_002168) 1-7 (1-323), 9-11 (361-453)

IKZF2 (NM_016260) 2-8 (1-527)

JAK2 (NM_004972) 3-7 (11-220), 7-17 (241-723), 17-25 (747-1133)

KIT (NM_000222) 1-16 (1-765), 17-21 (788-977)

KMT2A (NM_005933) 2-3 (145-456), 3-6 (476-1212), 7-12 (1218-1507), 13-22 (1526-1979), 23 (1985-2024), 24-27 (2032-2690), 27 (2710-2747), 27-36 (2798-3970)

KRAS (NM_004985) 2-5 (1-189)

MDM2 (NM_002392) 1-5 (1-120), 7-11 (143-498)

MPL (NM_005373) 1-3 (1-122), 4-9 (131-490), 11-12 (522-636)

MYD88 (NM_002468) 1-5 (10-310)

NOTCH1 (NM_017617) 1-3 (1-135), 4 (197-237), 5-6 (248-367), 8-11 (419-587), 11-12 (632-672), 13-14 (716-785), 16-17 (823-885), 18 (914-981), 19 (1034-1057), 21-22 (1109-1196), 23 (1215-1294), 24 (1304-1338), 25-26 (1373-1673), 27 (1701-1723), 28 (1737-1779), 29-31 (1795-1935), 31 (1965-1978), 32-34 (2012-2556)

NPM1 (NM_002520) 1-11 (1-295)

NRAS (NM_002524) 2 (1-37), 3-5 (77-190)

PTPN11 (NM_002834) 1-8 (1-299), 9-15 (312-594)

RUNX1 (NM_001754) 3-6 (20-205), 8-9 (269-435), 9 (460-481)

TET2 (NM_001127208) 3 (1-616), 3-11 (636-2003)

TP53 (NM_000546) 2 (1-25), 4-6 (41-224), 7-10 (234-367)

WT1 (NM_024426) 1 (25-104), 1-2 (126-257), 4-10 (291-518)

Note: Coverage by gene and codon(s) tested for adequate amplicons

A multiplex polymerase chain reaction (PCR) analysis for internal tandem duplications (ITD) and kinase domain (D835) mutations in FLT3 was performed on DNA isolated from bone marrow aspirate samples as previously described by our group {Luthra, 2014, Next-generation sequencing-based multigene mutational screening for acute myeloid leukemia using MiSeq: applicability for diagnostics and disease monitoring}. Briefly, fluorescently-labeled PCR primers were utilized to amplify targeted juxtamembrane domain and kinase domain sequences. PCR product sizes were determined using capillary gel electrophoresis on a 3100 genetic analyzer (Applied Biosystems, Foster City, CA). The presence of PCR fragment larger than the wild-type allele was considered to constitute ITD. For D835 mutations, PCR products were digested with the Eco RV restriction enzyme prior to capillary electrophoresis. The wild-type allele cut by this enzyme result in 2 fragments, whereas mutations at D835 alter the Eco RV recognition site and result in one fragment. The allelic frequency (AF) is determined as the area under the curve of the mutant allele divided by the sum of mutant and wild-type alleles. The analytical sensitivity of this assay is approximately 1% mutant allele in a background of wild-type allele (detailed methodology reported by Luthra, 2014, Next-generation sequencing-based multigene mutational screening for acute myeloid leukemia using MiSeq: applicability for diagnostics and disease monitoring}.

NGS was performed on clinical-grade CLIA-certified myeloid NGS Illumina MiSeq (Illumina, Inc., San Diego, CA, USA) platforms developed at MDACC, to evaluate hot-spot regions and/or the coding regions of genes frequently implicated in myeloid malignancies using a 28, 53 or 81 gene panel. A minimum quality score of AQ30 was required for a minimum of 75% of bases sequenced ensuring high-quality sequencing results. Variant calling was performed with Illumina MiSeq Reporter Software using human genome build 19 (hg 19) as a reference and reads were aligned using the Integrative Genomics Viewer (IGV, Broad Institute, MA, USA). For clinical reporting, a minimum sequencing coverage of x250 (bidirectional true paired-end sequencing) was required. The analytical sensitivity was established at 5% mutant reads in a background of wild-type reads. Matched germline DNA was not available for comparison.

Eight-color flow cytometry immunophenotyping (FCI) for measurable residual disease (MRD) was performed using a combination of the following 4 tubes: (1) CD7-FITC, CD33-PE, CD19 PerCP-Cy5.5, CD34-PE-Cy7, CD13-APC, CD38-BV421, CD45-V500; (2) HLADR-FITC, CD117-PE, CD4-PerCP-Cy5.5, CD34-PE-Cy7, CD123-APC, CD19-eF780, CD38-BV421, CD45-V500; (3) HLA-DR-FITC, CD36-PE, CD56-PerCP-Cy5.5, CD34-PE-Cy7, CD64-APC, CD19-eF780, CD14-V450, CD45-V500; and (4) CD5-FITC, CD2-PE, CD22-PerCP-Cy5.5, CD34-PE-Cy7, CD38-APC, CD19-eF780, CD15-V450, CD45-V500. All antibodies were obtained from Becton Dickinson (San Jose, CA) or eBioscience (San Diego, CA). Samples were acquired on FACSCanto II instruments (BD Biosciences, San Diego, CA). MRD was identified in comparison with the known patterns of antigen expression by normal maturing myeloid precursors and monocytes as previously described .[Jaso et al. 2014 BMT, and Shah et al. 2018 BMT]. At least 200,000 live events were acquired to achieve a minimum sensitivity of 10−4 (.01%). MRD was defined as a neoplastic blast population with an abnormal pattern of antigen expression deviating from normal regenerating myeloid progenitors or expressing leukemia associated immunophenotype (LAIP). The abnormal blast population was quantified as a percentage of total events.

**Supplement Table 3**: Cohort 1 (Frontline AML) CRc rates with sequential FLT3 inhibitors

|  |  | CRc rates, % | | | |
| --- | --- | --- | --- | --- | --- |
| FLT3i | **Regimen** | 1^st^ FLT3i | 2^nd^ FLT3i | 3^rd^ FLT3i | 4^th^ FLT3i |
|  | **Total n=96** | 43/56 (77%) | 10/32 (31%) | 2/8 (25%) | 0 |
| Midostaurin (n=10) | CCT (n=9) | 7/8 (87%) | NA | 0% | NA |
|  | LIT (n=1) | 1/1 (100%) | NA | NA | NA |
|  | Single agent (n=0) | NA | NA | NA | NA |
| Sorafenib (n=33) | CCT (n=26) | 18/23 (78%) | 2/3 (100%) | NA | NA |
|  | LIT (n=7) | 3/6 (50%) | 0/1 (0%) | NA | NA |
|  | Single agent (n=0) | NA | NA | NA | NA |
| Crenolanib (n=12) | CCT (n=2) | NA | 0/2 (0%) | NA | NA |
|  | LIT (n=1) | NA | 1/1 (100%) | NA | NA |
|  | Single agent (n=9) | NA | 1/7 (14%) | 0/2 (0%) | NA |
| Gilteritinib (N=8) | CCT (n=4) | 0/1 (0%) | 2/3 (66%) | NA | NA |
|  | LIT (n=3) | 2/2 (100%) | 0/1 (0%) | NA | NA |
|  | Single agent (n=1) | NA | 0/1 (0%) | NA | NA |
| Quizartinib (N=31) | CCT (n=1) | 1/1 (100%) | NA | NA | NA |
|  | LIT (n=20) | 11/13 (85%) | 3/6 (50%) | 0/1 (0%) | NA |
|  | Single agent (n=10) | 0/1 (0%) | 1/7 (14%) | 2/2 (100%) | NA |
| Others (n=2) | CCT (n=0) | NA | NA | NA | NA |
|  | LIT(n=0) | NA | NA | NA | NA |
|  | Single agent (n=2) | NA | NA | 0/2 (0%) | NA |

FLT3i: FLT3 inhibitor; CRc: Composite complete remission rate; LIT, low intensity chemotherapy; CCT, intensive cytotoxic chemotherapy; NA, not applicable

**Supplementary table 4A: Cohort 2 (R/R AML) CRc rates with subsequent FLT3 inhibitors**

|  |  | CRc % | | | |  |
| --- | --- | --- | --- | --- | --- | --- |
| FLT3i | **Regimen** | **1^st^ FLT3i** | **2^nd^ FLT3i** | **3^rd^ FLT3i** | **4^th^ FLT3i** |  |
|  | **Total (n=301)** | **(82/183) 45%** | **(19/89) 21%** | **(3/25) 12%** | **(0/4) 0%** |  |
| Midostaurin | Total (n=10) | (3/6) 50% | (1/4) 25% | NA | NA |  |
|  | CCT (n=1) | (1/1) 100% | NA | NA | NA |  |
|  | LIT (n=9) | (2/5) 40% | (1/4) 25% | NA | NA |  |
|  | Single agent (n=0) | NA | NA | NA | NA |  |
| Sorafenib | Total (n=89) | (24/52) 46% | (7/30) 23% | (1/7) 14% | NA |  |
|  | CCT (n=26) | (11/20) 55% | (0/4) 0% | (0/2) 0% | NA |  |
|  | LIT (n=50) | (12/26) 46% | (7/19) 37% | (1/5) 20% | NA | |
|  | Single agent (n=13) | (1/6) 17% | (0/7) 0% | NA | NA |  |
| Crenolanib | Total (n=66) | (6/30) 20% | (4/25)16% | (0/9) 0% | (0/2) 0% |  |
|  | CCT (n=15) | (2/5) 40% | (2/6) 33% | (0/3) 0% | (0/1) 0% |  |
|  | LIT (n=4) | (0/3)0% | (0/1) 0% | NA | NA |  |
|  | Single agent (n=47) | (4/22) 18% | (2/18) 11% | (0/6) 0% | (0/1) 0% |  |
| Gilteritinib | Total (n=13) | (3/4) 75% | (3/8) 37% | (1/1) 100% | N/A |  |
|  | CCT (n=0) | NA | NA | NA | NA |  |
|  | LIT (n=2) | (1/1) 100% | (0/1) 0% | NA | NA |  |
|  | Single agent (n=11) | (2/3) 67% | (3/7) 43% | (1/1) 100% | NA |  |
| Quizartinib | Total (n=105) | (46/85) 54% | (4/16) 25% | (1/4) 25% | NA |  |
|  | CCT (n=0) | NA | NA | NA | NA |  |
|  | LIT (n=48) | (25/39) 64% | (2/7) 28% | (1/2) 50% | NA |  |
|  | Single agent (n=57) | (21/46) 46% | (2/9) 22% | (0/2) 0% | NA |  |
| Others* | Total (n=18) | (0/6) 0% | (0/6) 0% | (0/4) 0% | (0/2) 0% |  |
|  | CCT (n=1) | (0/1) 0% | NA | NA | NA |  |
|  | LIT (n=0) | NA | NA | NA | NA |  |
|  | Single agent (n=17) | (0/5) 0% | (0/6) 0% | (0/4) 0% | (0/2) 0% |  |

* Others included AP24534 (n=1), CEP701 (n=1), E6201 (n=6), FLX925 (n=5), KW2449 (n=2), SAR103168 (n=1), SEL25 (n=1), TG02 (n=1)

FLT3i: FLT3 inhibitor; CRc: Composite complete remission rate; LIT, low intensity chemotherapy; CCT, intensive cytotoxic chemotherapy; NA, not applicable

**Supplementary Table 4B. CRc rates following quizartinib (1^st^ FLT3i) failure (n=30) in cohort 2**

|  | N=30 | CRc,N | CRc,% |
| --- | --- | --- | --- |
| Sorafenib | 18 | 4 | 22% |
| Single | 5 | 0 | 0% |
| combo | 13 | 4 | 31% |
| Crenolanib | 9 | 2 | 22% |
| Single | 6 | 1 | 17% |
| combo | 3 | 1 | 33% |
| Midostaurin | 2 | 0 | 0% |
| Single | 1 | 0 | 0% |
| combo | 1 | 0 | 0% |
| Gilteritinib | 1 | 0 | 0% |
| Single | 1 | 0 | 0% |

**Supplementary Table 4C. CRc rates following sorafenib (1^st^ FLT3i) failure (n=35) in cohort 2**

|  | N | CRc,N | CRc,% |
| --- | --- | --- | --- |
| Quizartinib | 12 | 2 | 25% |
| Single | 6 | 1 | 17% |
| combo | 6 | 2 | 33% |
| Crenolanib | 14 | 1 | 7% |
| Single | 10 | 0 | 0% |
| combo | 4 | 1 | 25% |
| Midostaurin | 2 | 1 | 50% |
| Single | 1 | 0 | 0% |
| combo | 1 | 1 | 100% |
| Gilteritinib | 5 | 2 | 40% |
| Single | 5 | 2 | 40% |
| E6201 | 2 | 0 | 0% |
| Single | 2 | 0 | 0% |

**Supplementary Table 4D.** **CRc rates following Midostaurin (1^st^ FLT3i) failure (n=8) in cohort 2**

|  | N | CRc,N | CRc,% |
| --- | --- | --- | --- |
| Single agent Quizartinib | 2 | 1 | 50% |
| Quizartinib + LIT | 1 | 0 | 0% |
| Single Agent Crenolinib | 3 | 1 | 33% |
| Single Sorafenib | 1 | 0 | 0% |
| Gilteritinib +CCT | 1 | 0 | 0% |

**Supplementary Table 5. Correlation of Assessments by MFC and PCR at the time of response**

|  | PCR- Negative (n=17) | PCR- Positive (n=58) | P Value |
| --- | --- | --- | --- |
| MFC- Negative (n=29) | 13 | 16 | 0.001 |
| MFC- Positive (n=46) | 4 | 42 | 0.001 |

**Supplementary table 6. Response rates based on FLT3-ITD, DMNT3A and NPM1 status**

| **Co-Mutations** | **N** | **CRc Rate** | **P value** |
| --- | --- | --- | --- |
| DNMT3A (n=56) |  |  |  |
| DNMT3A (yes) | 21 | 47% | 0.125 |
| DNMT3A (no) | 35 | 60% |  |
| NPM1 (N=54) |  |  |  |
| NPM1 (yes) | 23 | 43% | 0.303 |
| NPM1 (no) | 31 | 64% |  |
| DNMT3A + NPM1 (N=56) |  |  |  |
| DNMT3A + NPM1 (yes) | 13 | 46% | 0.446 |
| DNMT3A + NPM1 (no) | 43 | 58% |  |

**Incidence of pretherapy FLT3-D835 mutation with sequential FLT3i exposure (in cohort-2)**

We also observed an increasing incidence of pretherapy FLT3-D835 mutation with first, second and 3rd/4th FLT3i exposures, 18% (21 of 117), 32% (14 of 44) and 46% (6 of 13), respectively (P=0.056). Specifically among patients treated with Type II FLT3-inhibitors including quizartinib and sorafenib the incidence of FLT3-D835 mutations increased with first, second, and 3th/4th FLT3i exposures: 5% (5 of 96), 34% (12 of 35) and 50% (3 of 6), respectively (P=0.003). This is in line with expected mechanisms of secondary resistance.

**FLT3 allelic frequency in sequential FLT3-inhibitor settings in Cohort 2**

Pretherapy FLT3-ITD allelic frequencies were available in 52% (n=96) of the patients in cohort 2. Pretherapy median FLT3- ITD allelic frequency prior to the first FLT3i exposure (n=65) versus pretherapy FLT3-ITD allelic ratio prior to the second FLT3i exposure (n=25) versus pretherapy FLT3-ITD allelic ratio prior to the 3rd/4th FLT3i exposure (n=6) were 0.418, 0.435, and 0.411, respectively. These data suggest that FLT3-ITD appears to have remained as a driver mutation in sequential settings, which also likely explains why these patients were selected to be treated with FLT3 inhibitors sequentially.

**Supplementary Figure 1.**

**A. OS in patients with FLT3-ITD and DNMT3A co-mutated patients**


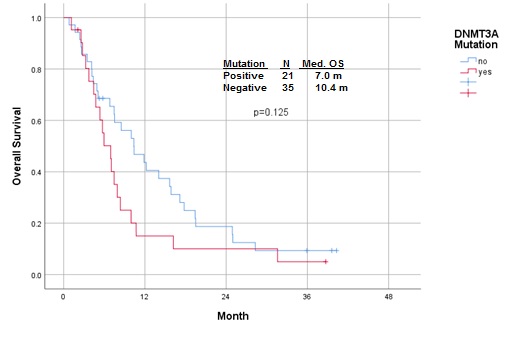


**B. OS in patients with FLT3-ITD and NPM1 co-mutated patients**


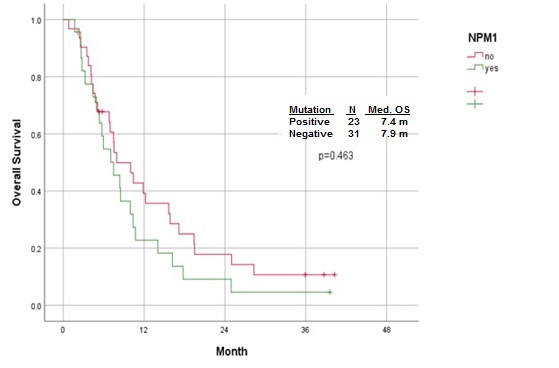


**C. OS in patients with triple (FLT3-ITD/NPM1/DNMT3A) mutated patients**


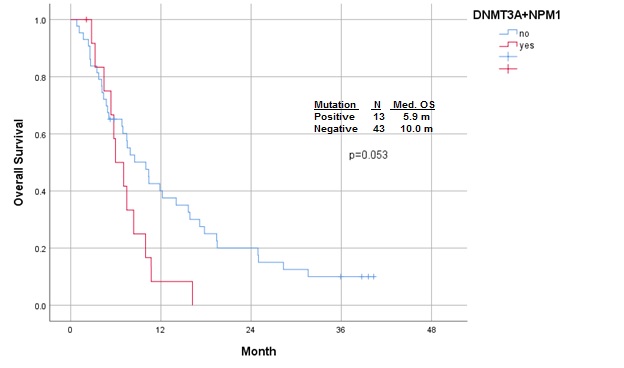


**Figure 1.**

We found no enhanced response rate in patients with DNMT3A mutations. In cohort-2, 56 patients with known FLT3-ITD had DNMT3A tested at baseline (prior to the 1st FLT3i exposure), and 21 (37%) harbored a DNMT3A co-mutation. The CRc rate was 47% in DNMT3A mutated patients (n=21) compared with 60% in DNMT3A non-mutated patients (n=35) (P = 0.367) and median OS was 7.0 months versus 10.4 months (P = 0.125) in DNMT3A mutated versus non-mutated patients (Supplementary table 6 and figure 1A).

In cohort 2, 54 patients with known FLT3-ITD had NPM1 tested at baseline (prior to the 1st FLT3i exposure), and 23 (43%) harbored an NPM1 co-mutation. The CRc rates and median OS were not significantly different in patients who had NPM1 co-mutations compared with patients without an NPM1 co-mutation (Supplementary table 6 and supplementary figure 1B). In cohort 2, 56 patients had both NPM1 and FLT3-ITD tested at baseline (prior to first FLT3i exposure) and 13 (23%) had NPM1 and DNMT3A co-mutation. Although CRc rates were not significantly different among those with NPM1 and DNMT3A commutations versus not, the median OS suggested a clear trend to inferior OS among patients who had NPM1 and DNMT3A co-mutations (Supplementary table 6 and supplementary figure 1C), although numbers are small to make a definite conclusion.

Of note, in our study cohort patients treated with various FLT3i based therapies with different backbone therapies; 37 quizartinib, 10 sorafenib, 7 crenolanib, 1 midostaurin, 1 gilteritinib (11 single-agent vs. 45 in combination with other regimens). This may make the co-mutational impact on outcome difficult to clearly assess in our series compared with those presented in the ADMIRAL and QUATUM-R datasets.
